# Retraction: Identification of the Sites of Tau Hyperphosphorylation and Activation of Tau Kinases in Synucleinopathies and Alzheimer’s Diseases

**DOI:** 10.1371/journal.pone.0325958

**Published:** 2025-06-06

**Authors:** The PLOS One Editors

Following the publication of this article and associated Expression of Concern [1,2], additional concerns were raised with results presented in Figs 1–3. Specifically,

- There appear to be vertical irregularities in the following panels:
  Fig 1A Thr205 and GAPDH
  ∘ Fig 1B Thr181
  ∘ Fig 1C **Thr212 and **Thr217
  ∘ Fig 1D *Ser202
  ∘ Fig 2A β-Syn and GAPDH
  ∘ Fig 2B γ-Syn
  ∘ Fig 3B GSK-3β
- There appear to be vertical and horizontal irregularities in the Fig 1D * Ser356 and * Ser409 panels.

The authors did not respond to editorial communications regarding the concerns above or the concerns summarised in [2]. In the absence of an author response and the underlying data, these issues cannot be resolved.

In light of the concerns with this article described above and in [2], which call into question the reliability and integrity of the published results, the *PLOS One* Editors retract this article.

DCM responded but expressed neither agreement nor disagreement with the editorial decision. VD, JHL, JC, JW, AO, CS, KS, EM, and AS either did not respond directly or could not be reached.

## References

[1] Duka V, Lee J-H, Credle J, Wills J, Oaks A, Smolinsky C, et al. Identification of the sites of tau hyperphosphorylation and activation of tau kinases in synucleinopathies and Alzheimer’s diseases. PLoS One. 2013;8(9):e75025. doi: 10.1371/journal.pone.0075025 24073234 PMC3779212

[2] PLOS ONE Editors. Expression of Concern: Identification of the Sites of Tau Hyperphosphorylation and Activation of Tau Kinases in Synucleinopathies and Alzheimer’s Diseases. PLoS One. 2023;18(12):e0295638. doi: 10.1371/journal.pone.0295638 38051714 PMC10697507

